# The analysis of inflammatory cell migration using primary neutrophils

**DOI:** 10.1186/1546-0096-13-S1-P136

**Published:** 2015-09-28

**Authors:** E Avci, YZ Akkaya Ulum, E Yilmaz, B Balci-Peynircioglu

**Affiliations:** 1 Hacettepe University, Medical Biology, Ankara, Turkey

## Introduction

Neutrophils are the primary defense of the host against pathogens. They remain in resting state in the circulation of the healthy individuals. When encountering cytokine or chemokine signals caused by pathogen products, neutrophils are activated and mobilized to the infected sites. The life span of neutrophils which have an essential role in terms of pathogenesis of auto-inflammatory diseases are very short. In consideration of this issue, it is restricted to study neutrophil migration *in vitro.* Hence, we developed a new approach on cell migration assays and neutrophil priming for long lasting research.

## Objectives

The aim of this study is to constitute a model for the analysis of inflammatory cell migration using primary neutrophils.

## Methods

Neutrophil cells were isolated from peripheral blood samples by using Lympholyte-poly solution. The purity and viability of the isolated neutrophil samples were assessed by May-Grundwald Giemsa staining and trypan blue exclusion, respectively. Following that, freshly isolated blood neutrophils were placed into glass-bottom chamber slides. Some of slides were treated with collagen in order to see the effect of extracellular matrix. Then, fMLP (N-formyl- Met-Leu-Phe), a classical chemoattractant for neutrophil was used to stimulate the cells for cell migration. The optimal chemoattractant dose was determined by imaging the polarization state of cells. Lastly, a modified Boyden chamber assay was used to quantitate chemotaxis rate of the neutrophils.

## Results

Neutrophil cells showed higher affinity to glass surface than plastic. The cells attached to the bottom of the slides were primed for long duration. We observed more cells in non-collagen slides when compared to collagen coated slides. Neutrophil cells were cultured for maximum 4 days under these conditions. The optimal fMLP dose was determined as 50μM for 30 minutes with 60% efficiency of neutrophil polarization visualized by actin staining. Quantitation of migrating cells using calcein-AM staining after Boyden chamber assay showed that 100nM fMLP for 24 hours was resulted in a high rate of migration.

## Conclusion

Most of *in vitro* experiments using primary neutrophils often fail to accomplish as a consequence of their short life span. We managed to keep neutrophil alive for 4 days. Furthermore, our results suggest a method for studying cell migration using primary neutrophils. The ability of longer neutrophil priming would enhance outputs of the researches in terms of studying molecular pathophysiology of auto-inflammatory diseases. Hereby, this study provides a novel approach to expand our knowledge of disease pathogenesis associated with neutrophil cells.

